# Bleeding Events During Anticoagulation After Acute Pulmonary Embolism: Real-Life Experience

**DOI:** 10.3390/medicina62071278

**Published:** 2026-07-02

**Authors:** Irina Pocienė, Brigita Lebednykienė, Jolita Račkauskienė, Vaida Averjanovaitė, Edvardas Danila

**Affiliations:** 1 Centre of Pulmonology and Allergology, Vilnius University Hospital Santaros Klinikos, 08406 Vilnius, Lithuania; brigita.lebednykiene@santa.lt (B.L.); jolita.rackauskiene@santa.lt (J.R.); vaida.averjanovaite@santa.lt (V.A.); edvardas.danila@santa.lt (E.D.); 2 Clinic of Chest Diseases, Immunology, and Allergology, Faculty of Medicine, Vilnius University, 03101 Vilnius, Lithuania

**Keywords:** pulmonary embolism, anticoagulation, bleeding, risk factors, long-term treatment

## Abstract

*Background and Objectives*: Pulmonary embolism (PE) is a potentially life-threatening disease. Although anticoagulant therapy reduces the risk of recurrent PE, it increases the risk of bleeding complications. Therefore, decisions regarding treatment duration are made individually, balancing recurrent venous thromboembolism (VTE) and bleeding risk. However, the optimal duration of anticoagulant therapy after acute PE remains challenging in clinical practice. The aim of this study was to evaluate bleeding rates during anticoagulant therapy and identify possible bleeding risk factors. *Materials and Methods*: A prospective study was conducted at a tertiary pulmonology center within a university hospital. A total of 201 consecutive patients (50.2% male) after a first episode of acute PE were included. Bleeding complications during anticoagulant therapy were recorded at follow-up visits and classified as major and minor. Potential risk factors associated with increased bleeding risk during anticoagulant therapy were analyzed. *Results*: During follow-up, 35 patients (17.4%) experienced bleeding complications, including 5 (2.5%) major and 30 (14.9%) minor bleeding events. Recurrent bleeding occurred in 6 patients (17.1%). The median time to first bleeding event was 3 months (IQR 1–7.5). Patients receiving extended anticoagulation beyond 6 months experienced more frequent bleeding events, predominantly non-major bleeding; however, bleeding incidence per patient-year did not differ significantly according to treatment duration (IRR 1.26, 95% CI 0.63–2.49). Other factors associated with increased bleeding risk included prior bleeding history, PE with no identifiable provoking factor, elevated B-type natriuretic peptide (BNP) levels and high early PE mortality risk at hospitalization. *Conclusions*: Bleeding during anticoagulant therapy after PE was frequent, but mostly non-major. More bleeding events (non-major) were observed among patients receiving longer anticoagulation, although this difference was attenuated after adjustment for anticoagulation exposure time. Prior bleeding, unprovoked PE, and markers of more severe PE were associated with increased bleeding risk. Findings suggest that prolonged anticoagulation is safe when clinically indicated, although regular reassessment of bleeding risk remains important.

## 1. Introduction

PE is a potentially life-threatening disease with an annual incidence of approximately 60 to 120 per 100,000 population [1]. Most patients with PE are treated with anticoagulants after hospital discharge. Historically, anticoagulant therapy after PE was usually prescribed for 3 months [2,3]. As more evidence became available, longer treatment durations, including 3–6 months or extended anticoagulation, began to be recommended due to the risk of recurrent PE [4,5]. The risk of recurrent VTE after discontinuation of anticoagulant therapy following the standard treatment period for PE is high: approximately 5% in the first year, reaching up to 30% at five years [6,7]. Extended anticoagulation can reduce the risk of recurrent VTE by 80 to 90% [8,9]. On the other hand, prolonged anticoagulation is associated with an increased risk of major bleeding, with an annual incidence of approximately 2–3% [10,11,12,13]. Reduced dose direct oral anticoagulants (DOACs) regimens for extended anticoagulation have been shown to lower bleeding risk [14,15]. However, in clinical practice, determining the optimal duration of anticoagulant therapy after a first episode of PE remains challenging. In each case, treatment decisions require careful balancing between the risks of VTE recurrence and bleeding [15,16].

The aim of this study was to evaluate bleeding incidence and identify potential risk factors associated with bleeding during anticoagulant therapy after acute PE.

## 2. Materials and Methods

### 2.1. Study Design

A prospective real-life study was conducted to evaluate bleeding incidence and associated risk factors in patients receiving anticoagulant therapy after acute PE. The study was performed at a tertiary pulmonology center at a university hospital. Patients were enrolled from 2015 onwards. Only patients with completed or available follow-up data were included in the analysis.

### 2.2. Participants

Adult patients (≥18 years) with newly diagnosed PE confirmed by computed tomography pulmonary angiography (CTPA) who received anticoagulant therapy were eligible for inclusion. Patients with recurrent PE, those unable to provide informed consent, and individuals who refused to participate were excluded from the study.

Baseline data, including patient demographics, comorbidities, concomitant medication use, PE severity, and risk of early mortality, laboratory parameters, type and dose of anticoagulant therapy, were collected to assess potential risk factors for bleeding during follow-up.

After the acute treatment phase, patients were prescribed anticoagulant therapy with DOACs, vitamin K antagonists (VKAs), or low-molecular-weight heparins (LMWHs) for outpatient treatment. The decision to reduce the dose of rivaroxaban or apixaban for extended treatment beyond 6 months was made individually, based on recurrence risk, bleeding risk, and residual thrombi on imaging.

### 2.3. Patient Follow-Up

Follow-up visits were scheduled at 1, 3, 6, and 12 months, with annual visits thereafter if continued monitoring was required after the first year following the acute PE episode. The number of follow-up visits for each patient depended on the duration of anticoagulant therapy, guided by PE risk factors, the time to thrombus resolution (assessed by CTPA or lung ventilation-perfusion (V/Q) scan), complications of anticoagulant therapy, and the risk of disease recurrence.

### 2.4. Assessment of Bleeding

At each follow-up visit, patient adherence to anticoagulant therapy, the occurrence of any bleeding events, hemoglobin concentration from a full blood count, and coagulation parameters (for patients receiving VKAs) were assessed. Bleeding events were identified during routine follow-up visits and classified according to the International Society on Thrombosis and Haemostasis (ISTH) criteria [17] by the study investigators (no independent adjudication committee was used). Major bleeding was defined as fatal bleeding, bleeding in a critical organ, or bleeding causing hemoglobin drop ≥20 g/L or transfusion ≥2 units. Clinically relevant non-major bleeding (CRNM) was bleeding that did not meet major criteria but required medical intervention or hospitalization. Minor bleeding was defined as any bleeding that did not meet the criteria for major or CRNM bleeding and did not require medical attention.

### 2.5. Ethics

This research was conducted in accordance with all relevant guidelines and procedures and was approved by the Vilnius Regional Biomedical Ethics Committee (No. 158200-13-652-210).

### 2.6. Statistical Analysis

All statistical analyses were performed using the R (v. 4.4.2) program package. The mean, standard deviation (SD), quartiles (Q1 and Q3), median and the available number of observations of the quantitative variables are presented. Categorical variables are presented as absolute counts and percentages. For statistical analysis, bleeding events were classified into non-major and major groups. The non-major bleeding category included both CRNM and minor bleeding events, because only two CRNM events were recorded, limiting a meaningful subgroup analysis. Recurrent bleeding episodes were not analyzed as independent events, and patients were classified according to the occurrence of at least one bleeding event during follow-up. Normality was tested using the Shapiro–Wilk test. To test hypotheses for comparison between two groups of quantitative variables, Student’s *t*-Test or the nonparametric Mann–Whitney U test was used as appropriate. To test hypotheses for between-group comparison of the categorical variables, Pearson’s Chi-Square or Fisher’s exact tests were used as appropriate. Cumulative bleeding incidence was estimated using the Kaplan–Meier method. Relative risks (RR) with 95% confidence intervals (CIs) were calculated using Fisher’s exact test to assess associations between categorical risk factors and bleeding outcomes. A *p*-value < 0.05 was considered statistically significant.

### 2.7. Use of GenAI

Generative AI(GPT 5.5) was used for generation of graphical elements and language editing.

## 3. Results

### 3.1. Study Population

**Figure 1:**
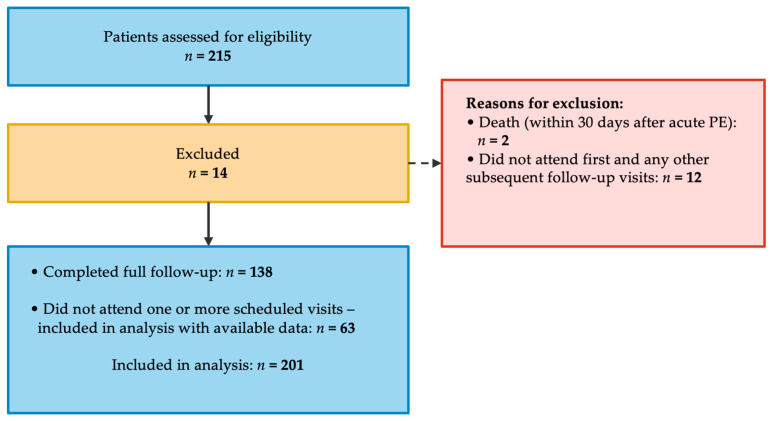
Study population flow chart.

A total of 215 consecutive patients were initially included in the study. Fourteen patients were excluded because they did not attend the first or any subsequent follow-up visits. Most of the remaining patients attended all scheduled visits, while those who missed some visits (most commonly the later or final visits) were still included in the final analysis using available data, resulting in a total of 201 patients (see Figure 1: Study population flow chart).

The study population comprised 101 men (50.2%) and 100 women (49.8%), with a median age of 65 years (interquartile range [IQR] 53–75). Most patients (n = 147, 73%) had no identifiable risk factor for PE (unprovoked PE). The majority were classified as having intermediate-high early mortality risk (n = 85, 43.1%). More detailed information about the baseline study population is provided in Table 1.

### 3.2. Anticoagulant Therapy

Most patients were treated with anticoagulants during hospitalization in accordance with guideline recommendations [5], considering mortality risk, comorbidities and socioeconomic factors, as DOACs were not reimbursed in the country at the beginning of the study. In-hospital thrombolysis was administered to 12 patients (6%).

After discharge, 161 patients (80%) were prescribed DOACs, most commonly rivaroxaban (n = 157, 98.1%), while 28 patients (14%) received warfarin, and 12 patients (6%) received low-molecular-weight heparins (LMWHs).

The median duration of anticoagulation for acute PE (complete thrombus resolution was confirmed by CTPA or V/Q scan) was 6 months (IQR 3–10). In some patients, anticoagulation was continued due to persistent thrombotic risk factors or other indications (e.g., deep vein thrombosis, atrial fibrillation). In these cases, the overall duration of anticoagulation during follow-up was longer, with a median of 8 months (IQR 6–15).

During the follow-up period, anticoagulant therapy was modified in 27 patients (13%). The most common reason for switching therapy was failure to achieve stable anticoagulation with warfarin (n = 12, 44.4%). Among patients treated with warfarin, INR was monitored at follow-up visits, with 40.9% of measurements within the therapeutic range, 45.5% below it, and 13.6% above it. Other reasons for switching included newly diagnosed cancer (n = 5, 18.5%), renal impairment (n = 2, 7.4%), and financial inability to continue treatment with DOACs (n = 2, 7.4%).

In 16 cases (8%), patients discontinued anticoagulant therapy without medical advice, despite an indication for continued treatment. The median time to temporary discontinuation was 4 months (IQR 2.8–7.2) after anticoagulation initiation, ranging from 1 to 24 months. The median duration of treatment interruption was 18 days (IQR 7–32.5), with a range of 2–120 days. In only 3 cases (18.8%), treatment interruption occurred due to patient concerns about bleeding. The remaining discontinuations occurred for the following reasons (2 cases each, 12.5%): failure to obtain a new prescription, financial constraints, discontinuation prior to planned invasive procedures (without switching to alternative anticoagulation), and discontinuation advised by a general practitioner. In 5 cases (31.3%), patients were unable to specify the reason for discontinuation of anticoagulation.

During follow-up, 57 patients (28.4%) died. Of these, 16 patients (28.1%) died within the first year of follow-up. No bleeding-related deaths were observed. The most common known cause of death was cancer (n = 16, 28.1%). Two deaths (3.5%) were due to recurrent PE, while the remaining deaths were related to other comorbidities, infectious diseases (such as pneumonia, sepsis) and stroke.

### 3.3. Bleeding Events

**Figure 2:**
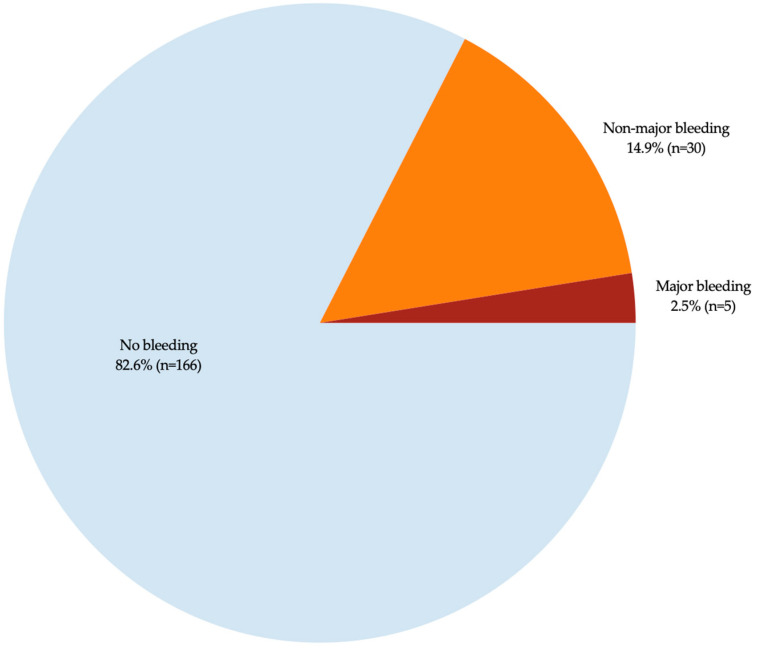
Frequency of bleeding events during anticoagulation therapy after PE.

Overall, 35 patients (17.4%) experienced bleeding complications during anticoagulation therapy, including 5 (2.5%) major and 30 (14.9%) minor bleeding events (see Figure 2. Frequency of bleeding events during anticoagulation therapy after PE). However, no bleeding-related deaths occurred during follow-up. Overall, 6 patients (17.1%) experienced recurrent bleeding, including 4 (11.4%) with two episodes and 2 (5.7%) with three episodes. In all cases, subsequent bleeding episodes occurred at the same anatomical site. The locations of major and non-major bleeding events are presented in Table 2.

The median time to first bleeding event was 3 months (IQR 1–7.5) after hospital discharge, with a range of 1–32 months. The cumulative incidence of bleeding events increased progressively over time, from 10.2% at 3 months to 13.5% at 6 months and 21.9% at 12 months of follow-up. Although major bleeding events tended to occur later than non-major events (median 5 months, IQR 2–6 vs. 3 months, IQR 1–8), no statistically significant difference in time to bleeding was observed between the two groups (*p* = 0.51).

No risk factor for bleeding was found in the majority of cases (n = 27, 77.1%). In cases where a predisposing comorbidity or risk factor was identified, gynecological diseases accounted for 4 cases (11.4%), including uterine fibroids (n = 2), endometriosis (n = 1), and endometritis (n = 1). Warfarin overdose was identified in 2 cases (5.7%), while hemorrhoids and urethral stricture (post-radiotherapy) each accounted for one case (2.9%).

### 3.4. Bleeding Risk Factors

**Figure 3:**
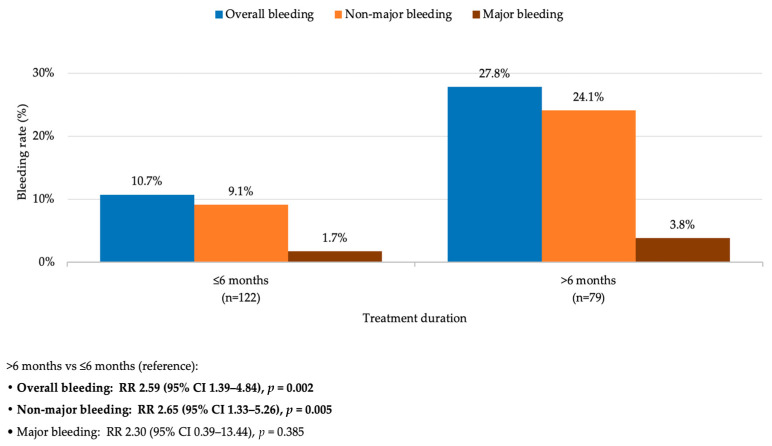
Duration of anticoagulation and bleeding events. RR = *relative risk*; CI = *confidence interval.* No significant difference in bleeding rate was observed between the ≤3-month (n = 52) and 3–6-month (n = 70) treatment groups.

Extended anticoagulation beyond 6 months was associated with significantly more bleeding events compared with ≤6 months of treatment (27.8% vs. 10.7%; RR 2.59, 95% CI 1.39–4.84; *p* = 0.002). This difference was mainly due to non-major bleeding events in the extended treatment group (24.1% vs. 9.1%; RR 2.65, 95% CI 1.33–5.26; *p* = 0.005). No significant differences in bleeding rates were observed between the 3–6 month and 3-month treatment groups (11.4% vs. 9.8%, RR 1.17, 95% CI 0.40–3.36; *p* = 1.000). Major bleeding rates did not differ significantly across treatment duration groups (see Figure 3. Duration of anticoagulation and bleeding events). To account for differences in anticoagulation exposure time, bleeding incidence rates were also calculated per 100 patient-years of anticoagulation. The overall bleeding incidence was 18.8 per 100 patient-years. The incidence rate was 16.4 per 100 patient-years among patients treated for ≤6 months and 20.6 per 100 patient-years among those treated beyond 6 months, corresponding to an incidence rate ratio of 1.26 (95% CI 0.63–2.49).

Bleeding events were also more frequent in patients with unprovoked PE compared to those with provoked PE (21.1% vs. 7.4%; RR 2.85, 95% CI 1.05–7.69; *p* = 0.022).

Patients with high early mortality risk had more pronounced right ventricular dysfunction, shown by significantly higher BNP levels compared with those at lower risk (median 723 ng/L, IQR 310–1133 vs. 139 ng/L, IQR 41–435; *p* = 0.021). Patients with major bleeding also had higher BNP levels at admission than those without bleeding (median 963 vs. 136 ng/L; IQR 606–1441 vs. 40–443; *p* = 0.018). In addition, patients with high early mortality risk at hospitalization experienced more bleeding events during follow-up compared with those at lower risk categories (57.1% (4/7) vs. 16.1% (31/192), *p* = 0.019). Initial treatment with thrombolysis was not associated with bleeding outcomes during follow-up.

A history of bleeding prior to PE was associated with bleeding during follow-up (100% (2/2) vs. 16.6% (33/199); *p* = 0.030). However, this finding should be interpreted with caution, given the very small number of patients with a prior history of bleeding (n = 2).

Other comorbidities, concomitant medications potentially increasing bleeding risk, and other laboratory parameters (including renal function) were not associated with bleeding events.

## 4. Discussion

In this prospective observational study, we evaluated the incidence of bleeding complications and their associated risk factors during anticoagulation after a first episode of PE. Our main findings, discussed further below, were as follows. Bleeding complications were common, although most were clinically non-significant. The median time to first bleeding event was relatively short, reaching 3 months after initiation of anticoagulation. Bleeding frequency was higher among patients receiving longer anticoagulant treatment, mainly due to non-major bleeding events. Finally, prior bleeding history and markers of more severe PE, particularly elevated BNP levels reflecting right ventricular dysfunction, were associated with increased bleeding risk.

The overall bleeding rate in our study was 17.4%, which was slightly higher compared with previous studies reporting overall bleeding rates of approximately 8 to 15% [12,13,18]. However, this finding was mainly related to a greater proportion of non-major, clinically insignificant bleeding events. The prospective nature of our study and regular follow-up visits may have increased the detection of minor bleeding events. Major bleeding events accounted for 2.5% of all bleeding events, which is consistent with previously published data [12,13,18,19]. Importantly, major bleeding events in our study frequently occurred in patients with comorbidities associated with increased bleeding risk, mainly gynecological diseases. No bleeding-related deaths or critical-site bleeding events were observed in our study. This could be partially explained by regular patient follow-up and detailed patient education on early recognition and management of bleeding complications.

Our results also showed that patients receiving extended anticoagulation beyond 6 months experienced a higher overall frequency of bleeding events. Importantly, this increase was mainly due to non-major bleeding, whereas major bleeding rates did not differ significantly according to treatment duration. However, when bleeding incidence was adjusted for anticoagulation exposure time, the difference between groups was no longer statistically significant. This suggests that the higher bleeding frequency observed during extended anticoagulation may be largely explained by longer treatment duration and greater cumulative exposure to anticoagulants. These findings are consistent with previous studies demonstrating an increasing cumulative risk of bleeding during prolonged anticoagulation [20,21].

The median time to the first overall bleeding event in our study was 3 months. It is in line with previous studies demonstrating that anticoagulant-related bleeding risk is highest during the initial months of treatment [22]. In contrast to these studies, our findings showed that major bleeding events tended to occur later than non-major bleeding events, although the difference was not statistically significant (median 5 vs. 3 months). This finding likely reflects the characteristics of patients with major bleeding, as several events occurred in women with gynecological diseases associated with an increased bleeding risk. In these patients, the treatment of the underlying disease was postponed during the early post-PE period, which may have prolonged the underlying bleeding risk and contributed to the later occurrence of major bleeding events.

In our study, unprovoked PE was associated with more frequent bleeding events. Traditionally, unprovoked PE is associated with an increased risk of recurrent VTE and frequently requires extended or indefinite anticoagulant therapy [4,21,23]. Therefore, the higher bleeding frequency observed in these patients may partly be explained by the longer duration of anticoagulant treatment compared with patients with transient provoking factors.

Notably, our findings showed that patients with high early PE mortality risk and elevated BNP levels, likely reflecting right ventricular dysfunction, experienced more bleeding events during follow-up. Evidence linking PE severity and markers of right ventricular dysfunction with long-term bleeding risk remains limited [5,24]. In our study, echocardiographic data were not available for all patients, and other markers of right ventricular dysfunction were not systematically analyzed. Although patients with more severe PE may differ in comorbidity profile, and treatment characteristics, none of these factors were significantly associated with bleeding risk in our study. Therefore, the mechanisms underlying our findings are not fully clear. Further studies are needed to better understand these findings. Nevertheless, these findings should be interpreted cautiously, given the limited number of patients in the high-risk PE group.

Although based on a small number of patients, a history of bleeding prior to PE was associated with an increased risk of bleeding events during follow-up in our study. Previous studies have also identified prior bleeding as an established bleeding risk factor during anticoagulant therapy [25,26]. Nevertheless, this finding should be interpreted cautiously given the limited number of patients with a prior bleeding history in our study.

Surprisingly, other established bleeding risk factors, including age, sex, concomitant medication use, comorbidities, and laboratory findings [14], were not associated with increased bleeding frequency in our study. This may reflect the limited number of major bleeding events and the predominance of non-major bleeding.

Less than half of warfarin-treated patients in our study had INR values within the therapeutic range. Although suboptimal INR control has been associated with both bleeding and thromboembolic complications [27], we did not specifically evaluate the relationship between INR control and clinical outcomes.

Bleeding risk assessment in patients with VTE continues to evolve and may be influenced by biological factors that are not routinely incorporated into current prediction models. A recent study suggested that low low-density lipoprotein cholesterol levels may be associated with increased bleeding risk in patients with VTE [28]. However, this variable was not available in the present study.

## 5. Strengths and Limitations

The strengths of this study include its prospective design, regular follow-up visits, and a real-world PE population. This enabled close monitoring of bleeding events during anticoagulant therapy, including clinically less significant bleeding episodes.

This study also has several limitations. First, the relatively small sample size and the limited number of bleeding events reduced the statistical power to detect associations with less frequent or established bleeding risk factors, increasing the possibility of type II error. In addition, reliable multivariable analysis could not be performed. Therefore, potential confounding between associated risk factors cannot be excluded. Second, most patients received rivaroxaban; therefore, the bleeding risk between different anticoagulants could not be reliably compared. Finally, the risk of bleeding with reduced-dose DOAC therapy during extended anticoagulation could not be evaluated properly, as only a small number of patients received reduced doses, since this treatment strategy was not yet widely used at the beginning of the study.

## 6. Conclusions

Overall bleeding complications during anticoagulant therapy after PE were common, but mostly clinically non-significant. Major bleeding events were rare and mainly occurred in patients with comorbidities associated with increased bleeding risk. Patients receiving extended anticoagulation experienced more frequent bleeding events, predominantly non-major bleeding. Prior bleeding history, unprovoked PE, and higher early PE mortality risk at hospitalization, mainly reflecting right ventricular dysfunction, were associated with increased bleeding risk. However, considering the high risk of recurrent VTE after anticoagulation discontinuation, these findings support extended anticoagulation when clinically indicated, with regular assessment of bleeding risk during follow-up.

## Figures and Tables

**Table 1:** Baseline characteristics of the study population stratified by bleeding status.

Variable	Total (n = 201)	No Bleeding (n = 166)	Bleeding (n = 35)	*p*
Demographic characteristics				
Male sex, n (%)	101 (50.2%)	79 (47.6%)	21 (60.0%)	0.198
Age (years), median (IQR)	65 (53–75)	65 (55–76)	63 (49–75)	0.747
BMI (kg/m^2^), median (IQR)	29.7 (26.0–35.0)	29.7 (26.0–34.9)	30.1 (25.1–35.2)	0.916
Ever smoker, n (%)	55 (27.4%)	47 (28.3%)	8 (22.9%)	0.677
PE characteristics				
Unprovoked PE, n (%)	147 (73.1%)	116 (69.9%)	31 (88.6%)	**0.022**
Provoked PE, n (%)	54 (26.9%)	50 (30.1%)	4 (11.4%)	**0.022**
Malignancy (active)	18 (33.3%)	18 (10.8%)	0 (0.0%)	**0.047**
Trauma	14 (25.9%)	13 (7.8%)	1 (2.9%)	0.471
Surgery	10 (18.5%)	10 (6.0%)	0 (0.0%)	0.215
Hormonal/obstetric factors	9 (16.7%)	6 (3.6%)	3 (8.6%)	0.192
Immobilization	2 (3.7%)	2 (1.2%)	0 (0.0%)	1.000
Thrombophilia	1 (1.9%)	1 (0.6%)	0 (0.0%)	1.000
PESI score, median (IQR)	90 (71–106)	90 (73–105)	93 (59–112)	0.770
PE severity and risk of early death, n (%)				
Low	49 (24.6%)	43 (25.9%)	6 (17.1%)	0.386
Intermediate-low	60 (30.2%)	52 (31.3%)	8 (22.9%)	0.417
Intermediate-high	85 (42.7%)	68 (41.0%)	17 (48.6%)	0.454
High	7 (3.6%)	3 (1.8%)	4 (11.4%)	**0.019**
Comorbidities				
Arterial hypertension, n (%)	118 (58.7%)	97 (58.4%)	21 (60.0%)	1.000
Diabetes mellitus, n (%)	30 (14.9%)	24 (14.5%)	6 (17.1%)	0.794
Malignancy (current or previous), n (%)	27 (13.4%)	24 (14.5%)	3 (8.6%)	0.428
Atrial fibrillation, n (%)	23 (11.4%)	20 (12.0%)	3 (8.6%)	0.772
Heart failure, n (%)	18 (9.0%)	18 (10.8%)	0 (0.0%)	**0.047**
Chronic kidney disease, n (%)	16 (8.0%)	11 (6.6%)	5 (14.3%)	0.163
Anemia, n (%)	16 (8.0%)	12 (7.2%)	4 (11.4%)	0.488
Prior major bleeding, n (%)	2 (1.0%)	0 (0.0%)	2 (5.7%)	**0.030**
Other comorbidities , n (%)	74 (36.8%)	–	–	–
Laboratory findings at admission				
D-dimer (µg/L), median (IQR)	2950 (1610–5795)	2845 (1551–5584)	3425 (1823–6368)	0.352
BNP (ng/L), median (IQR)	141.8 (42.1–445.0)	136 (40–443)	176 (84–487)	0.368
Creatinine (µmol/L), median (IQR)	82 (71–102.5)	80 (71–100)	91 (72–112)	0.223
Urea (mmol/L), median (IQR)	6.4 (5.3–8.1)	6.7 (5.3–8.4)	5.5 (4.2–6.2)	0.066
Hemoglobin (g/L), median (IQR)	137 (125–145)	137 (125–145)	137 (125–147)	0.581
Platelets (×10^9^/L), median (IQR)	199 (165–261)	198 (165–266)	205 (169–231)	0.898
CRP (mg/L), median (IQR)	23.7 (8.8–65.0)	24.6 (8.4–62.5)	21.3 (13.1–68.0)	0.787
Use of concomitant medications affecting hemostasis				
Antiplatelet agents, n (%)	18 (9.0%)	14 (8.4%)	4 (11.4%)	0.525
Anticoagulants (prior to PE), n (%)	7 (3.5%)	7 (4.2%)	0 (0.0%)	0.608
NSAIDs (regular use), n (%)	11 (5.5%)	9 (5.4%)	2 (5.7%)	1.000

Classification of PE severity and risk of early (in-hospital or 30-day) death according to the 2019 ESC PE guidelines [5]. Abbreviations: BMI—body mass index, PE—pulmonary embolism, PESI—pulmonary embolism severity index, BNP—B-type natriuretic peptide, CRP—C-reactive protein, NSAIDs—non-steroidal anti-inflammatory drugs, IQR—interquartile range.

**Table 2:** Locations of major and non-major bleeding.

Bleeding Location	Bleeding Events (n = 35)	Non-Major (n = 30)	Major (n = 5)
Urogenital	14 (40.0%)	11 (36.7%)	3 (60.0%)
Nasal	7 (20.0%)	7 (23.3%)	—
Gastrointestinal	4 (11.4%)	2 (6.7%)	2 (40.0%)
Gingival	4 (11.4%)	4 (13.3%)	—
Skin	3 (8.6%)	3 (10.0%)	—
Ocular	3 (8.6%)	3 (10.0%)	—

## Data Availability

The raw data supporting the conclusions of this article will be made available by the authors on request.

## Abbreviations

The following abbreviations are used in this manuscript:

BNP	B-type natriuretic peptide
BMI	Body mass index
CI	Confidence interval
CRNM	Clinically relevant non-major bleeding
CRP	C-reactive protein
CTPA	Computed tomography pulmonary angiography
DOAC(s)	Direct oral anticoagulant(s)
ESC	European Society of Cardiology
INR	International normalized ratio
IRR	Incidence rate ratio
IQR	Interquartile range
ISTH	International Society on Thrombosis and Haemostasis
LMWH(s)	Low-molecular-weight heparin(s)
NSAIDs	Non-steroidal anti-inflammatory drugs
PE	Pulmonary embolism
PESI	Pulmonary embolism severity index
Q1, Q3	First and third quartiles
RR	Relative risk
SD	Standard deviation
VKA(s)	Vitamin K antagonist(s)
V/Q	Ventilation-perfusion (scan)
VTE	Venous thromboembolism
